# Cystic encapsulated papillary carcinoma with ductal carcinoma in situ in the male breast: a clinicopathologic feature with a diagnostic challenge: A case report and review of literature

**DOI:** 10.1097/MD.0000000000034388

**Published:** 2023-11-03

**Authors:** Bo Wang, Li Wang, Zhenya Zhao, Xin Xu

**Affiliations:** a Department of Pathology, Xingtai, P. R. China; b Breast Surgery, Xingtai People’s Hospital, Hebei Medical University Affiliated Hospital, Xingtai, P. R. China.

**Keywords:** breast, clinicopathologic features, cystic, encapsulated papillary carcinoma, immunophenotype, male

## Abstract

**Rationale:**

Encapsulated papillary carcinoma (EPC) is a rare subset of breast carcinoma accounting for 0.5% to 2.0% of all patients with breast cancer and occuring mostly in postmenopausal women. It is even rarer in male breast cancer, and male EPC has only been reported in few cases. EPC has a distinctive histological pattern and a better prognosis compared with other types of breast carcinoma. Compared to the previously reported EPC cases, the lesion was unusually cystic made the diagnosis challenging. Accordingly, herein, we describe a rare case of EPC was unusually cystic in an elder male breast, associated with ductal carcinoma in situ (DCIS), along with an indepth literature discussion, and then to improve our understanding more about this uncommon tumor and further to provide more experience to treat this disease.

**Patient concerns:**

A 73-year-old man noticed a slowly enlarging mass in the right breast 1 year ago and sought medical attention. The patient presented with a right breast mass of 1-year duration and bloody nipple discharge in the first couple of days. The medical history was unremarkable.

**Diagnoses:**

Physical examination, an elastic hard, smooth and movable 4-cm lesion was palpated below the right papilla. On the sonography, a well-defined predominantly cystic-solid tumor of 3.6 × 2.3 cm was confirmed. Postoperative pathological and immunohistochemical examinations of the surgical specimens revealed a final diagnosis of breast EPC with DCIS.

**Interventions:**

The patient underwent surgery. A diagnosis of “a little papillary neoplasm of the breast with epithelial atypia and hypertrophy in the fibrous cystic wall” was made by the frozen section. Further, total mastectomy was performed.

**Outcomes:**

The operation was successful. Then the male patient recovered completely, did not require any additional treatment and continued to do well on postsurgical mammary surgical clinic visits. The patient had been followed-up regularly for 2 years after surgery; he did not experience any complications and remained disease-free.

## 1. Introduction

Encapsulated papillary carcinoma (EPC) of the breast is a rare subtype breast carcinoma that accounts for 0.5% to 2% of all breast cancers and occuring mostly in postmenopausal women.^[1]^ It was first reported in 1969 by McKittrick as a ductal carcinoma in situ (DCIS) variation and presented as subareolar masses and/or complained of nipple bloody discharge. Hill found that the myoepithelial cells of the tumor were missing or focal present in 2005 by immunohistochemical staining, suggesting that it was an indolent variant of breast invasive carcinoma with slowly developing form and expansive potential, so it might be a progressive lesion between carcinoma in situ and invasive carcinoma; meanwhile, he first proposed the name of EPC to describe the lesion more correctly.^[2]^ EPC is also referred to by several synonyms: intracystic papillary carcinoma, encysted papillary carcinoma, and intracystic carcinoma not otherwise specified. EPC is a special independent subtype of papillary breast lesion according to the World Health Organization classification of breast tumors (2012), which is divided into EPC and EPC with invasive type. An invasive component is generally in the form of conventional invasive ductal carcinoma (the synonym: non-special type invasive breast carcinoma, IBC-NST). EPC can exist alone, actually, it usually presents with a low to intermediate nuclear grade DCIS. Additionally, the prognosis is excellent in the both absence of and association with invasiveness.^[3]^ There are a series of challenges in diagnostic work, although it has distinctive histological pattern, especially the rare EPC was unusually cystic, that was easily confused with breast cyst, thereby causing misdiagnosis prior to or after surgery. Accordingly, it is important to be familiar with features of diagnosis and differential diagnosis of EPC, which can help to make the correct diagnosis; awareness of this entity, familiarity with the associated clinical features and recognition of the cellular morphological features of EPC, and then can help doctors avoid certain pitfalls in the diagnostic and treatment process. Accordingly, herein, we present a fairly rare case of EPC was unusually cystic occurring in a 73-years-old male patient, and review the literature has been published. The descriptions of EPC in this case and the review features based on published cases, should add to pathologists knowledge of diagnosis, can help clinicians improve the understanding of this rare disease, and further avoid inappropriate treatment.

## 2. Case report

A 73-year-old man entered the hospital due to discovering a slowly enlarging mass for 1 year in the right breast. The patient’s medical history was unremarkable. This case was treated in accordance with the ethical standards of Xingtai People’s Hospital.

**Figure 1.**
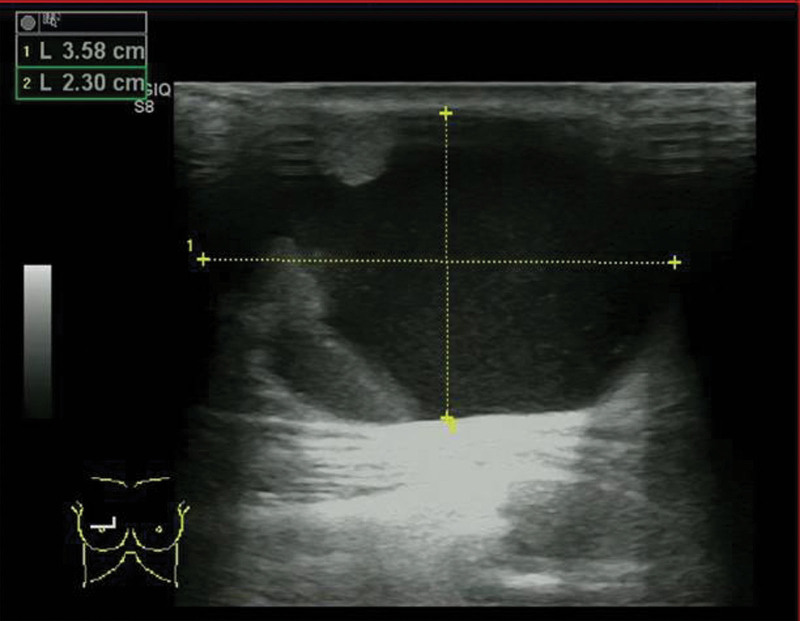
On mammary ultrasonography, a well-defined predominantly cystic-solid lesion of 3.6 × 2.3 cm was confirmed immediately below the right papilla (BI-RADS grade 3); a solid areas with a gradual rise from the cyst wall was confirmed within the cyst.

Physical examination, an elastic hard, smooth and movable 4-cm lesion was palpated below the right papilla. There were no skin lesions, and swollen lymph nodes were not palpated. On mammary ultrasonography, a well-defined cystic-solid lesion that 3.6 × 2.3 cm was confirmed immediately below the right papilla (BI-RADS grade 3); a 1.5 × 1 cm solid areas with a gradual rise from the cyst wall was confirmed within the cyst (Fig. 1). The patient underwent breast tumor resection for the fast-frozen pathology. A diagnosis of “a little papillary neoplasm of the breast with epithelial atypia and hypertrophy in the fibrous cystic wall with a little DCIS” was made by the frozen section. Further, total mastectomy was performed. The operation was successful. Then the patient recovered completely and continued to do well on postsurgical thoracic surgical clinic visits.

**Figure 2.**
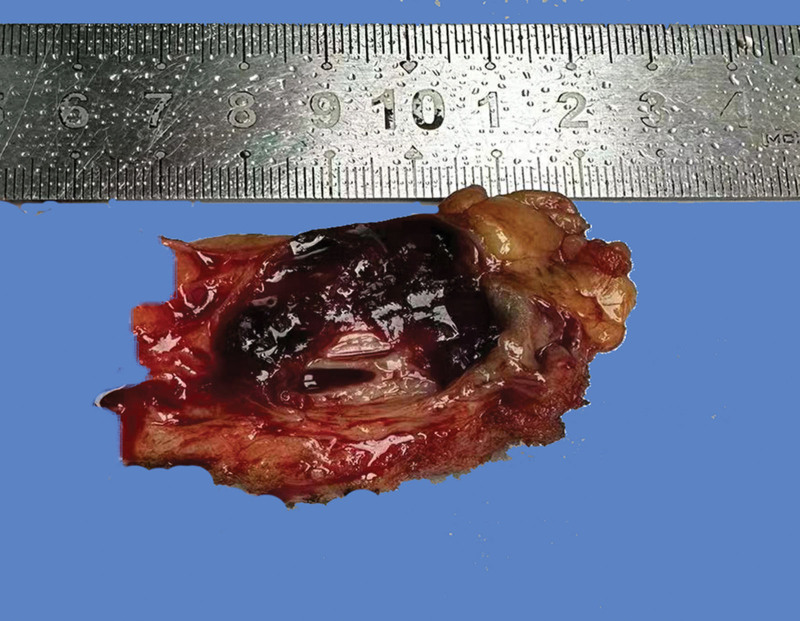
On gross examination, a well-circumscribed and entirely cystic lesion lined by a hemorrhagic thick fibrotic wall was found in the parenchyma of the resected breast tissue. The inner wall of the cyst was dark red.

**Figure 3.**
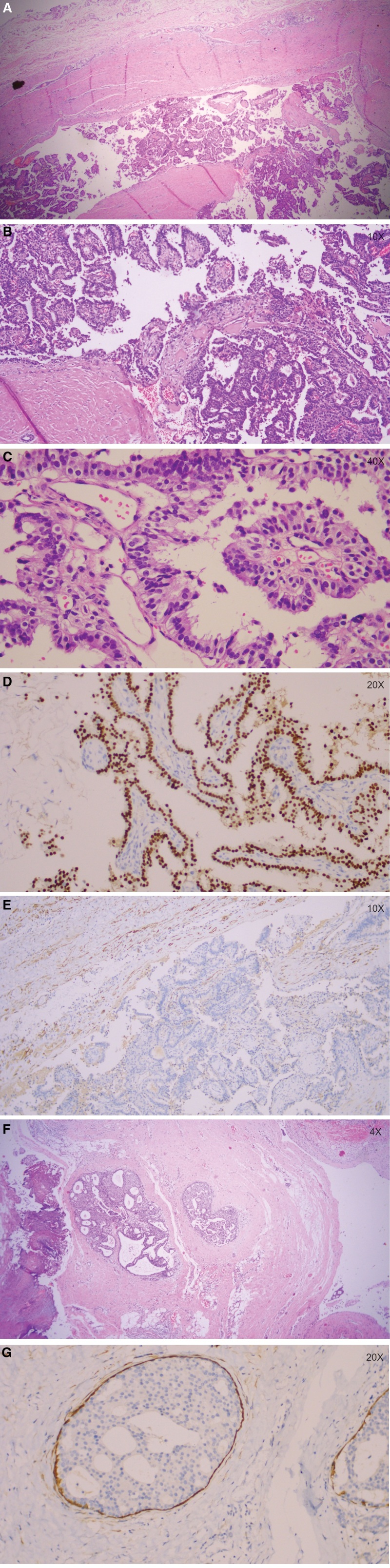
EPC associated with conventional DCIS. A: H&E sections at 25× magnification showing EPC composed of a thick fibrous capsule and a few sections with papillary carcinoma were identified. B: H&E sections at 200× magnification showing the tumor with slender fibrovascular stroma formed intraluminal arborization and covered by atypical solid, cribriform and micropapillary proliferative epithelium. C: H&E sections at 400× magnification showing tumor cells with mild to medium atypia around the delicate cores. D: 200× magnification, ER immunostain is strong diffuse nuclear staining in tumor cells. E:100× magnification, calponin immunostain is absent in both the cores and the periphery. F: H&E sections at 40× magnification showing cystic EPC (the right) associated with DCIS (the left). G: 200× magnification, Calponin immunostain is present at the periphery of DCIS. DCIS = ductal carcinoma in situ, EPC = encapsulated papillary carcinoma, ER = estrogen receptor.

The resected specimen was sent for histopathologic examination. On gross examination, a well-circumscribed and entirely cystic lesion lined by a hemorrhagic thick fibrotic wall was found in the parenchyma of the resected breast tissue (Fig. 2). The mass measured 3.5 × 2.5 × 1 cm, with a wall thickness of about 0.2 to 0.3 cm on inspection; the content was not observed in the cyst. The entire cyst wall was examined under microscopy; only a few sections with papillary carcinoma were identified (Fig. 3A), which with slender fibrovascular stroma and covered by atypical solid, cribriform and micropapillary proliferative luminal epithelium with mildly to moderately atypical nuclear and rare mitoses, there was no evidence of necrosis (Figs. 3B–C). A myoepithelial layer is absent both in papillary structures and in the capsule. The lesion was confined to the cyst wall. Immunohistochemistry showed the tumor was diffusely strongly positive for estrogen receptor (ER) (Fig. 3D), and about 20% of tumor cells were positive for progesterone receptor, while her-2 protooncogene expression was negative and ki-67 index was 20%; negative for CK5/6, P63 and calponin (Fig. 3E). Immunochemical analysis displayed in the lack of myoepithelial layer both in the center of fibrovascular cores and at the periphery of the tumor. Meanwhile, low grade DCIS in the nearby breast tissue has been discovered in this case (Figs. 3F–G). So, a diagnosis of EPC associated with DCIS was made. The patient has been followed-up regularly and has remained asymptomatic for 2 years subsequent to surgery.

## 3. Discussion

EPC of the male breast is an exceedingly rare carcinoma, especially when the EPC was unusually cystic, which was an unusual feature making the diagnostis especially challenging. EPC was divided into 3 subtypes: pure EPC, EPC associated with DCIS, and EPC associated with invasive carcinoma.^[4]^ It primarily affects postmenopausal women and usually is diagnosed at an average age of 67 years.^[3,5]^ However, it has been reported that EPC can occasionally affect younger patients. The average age of the patients suffered from EPC with invasion was 59.3 years, which is also considered relatively young. Rarely, EPC can occur in male patients, Male patients cover about 2% to 7% of EPC cases.^[6,7]^ The features of EPC including growth slower, better prognosis, rare local recurrence and distant metastasis are observed.^[8,9]^ In our study, the patients is a aging male and has a better prognosis. There were no distant metastasis or deaths by EPC during the follow-up periods.

Usually the EPC manifests as a painless lump in the breast, that could be present for several years. Clinically, EPC usually presents like a benign tumor that was asymptomatic and was found by screening mammography. A common symptom is bloody nipple discharge. Clinical and radiological findings of EPC are not very specific. Fine needle aspiration may be useful. A core needle biopsy may help distinguish benign and malignant papillary lesions. But it has a low accuracy for differentiation of invasive and noninvasive tumors,^[10]^ especially in the case of unusually cystic EPC, for its limitations of sampling, which is the major reason of false and uncertain diagnosis. There are authors propose surgical excision of such lesions for obtain the complete mass, sometimes without a biopsy.^[11]^ A surgical excisional biopsy is recommended for papillary lesions after a core needle biopsy, because of invasion is usually found in the peripheral part of the tumor; furthermore, EPC often be associated with DCIS.

EPC originates from the ductal epithelium, the components grow in a fragile solid and/or papillary lesion pattern surrounded by a capsule, that formed solitary nodule or multiple nodules with gray-red/gray-pink appearance, expansive growth and well-circumscribed. In the histopathological appearance, the tumor is comprised of cellular islands, which were composed of cells arranged in solid, mesh and/or micropapillary structures, with branches of fibrovascular cores. These tumor cells are small and monotonous with a low to moderate nuclear grade and rare mitoses in the majority of case. The cellular proliferations in the tumor nodule are homogenous. It is one of the main points in the diagnosis of EPC that lack of myoepithelial layer both in the center of papillary fibrovascular cores and at the periphery.^[12]^ Although many studies have shown that EPC mostly lack myoepithelial layer, both in the cores and at the periphery, they are still considered as inert invasive carcinoma. It is of utmost importance to recognize the EPC associated with invasive carcinoma as we found invasion in a high percentage of cases in studies. The size of invasive carcinoma varied, the type of invasive carcinoma is multifocal and mainly is invasive ductal carcinoma. EPC is usually a large size tumor (mean 2 cm) located within a large cystic duct, and is rare unusually cystic, that is the major reasons of false diagnosis as a benign cyst of breast when lacked of prudence and awareness surrounding the issue. Therefore, awareness of this unusual feature, repeat biopsy when the pathology result is discordant, and extensive sampling of the lesion are essential for making the correct diagnosis.

Myoepithelial markers- calponin, p63, CK5/6 are negative or only a few cells were positive in the papillary fiber axes and peripheral capsule wall. EPC usually express a luminal phenotype, that positive for ER and progesterone receptor but negative for HER2.^[13]^ Ki-67 has been used as a routine detection index for the diagnosis, prognosis and postoperative treatment of a variety of malignant tumors.^[14]^ It is commonly known that the high expression of Ki-67 is related to the increased risk of recurrence and poor prognosis. In this study, the Ki-67 index is about 20%, showed that at a lower expression level (index < 30%), indicating that the growth rate is relatively slow.^[15]^ But in cases of EPC associated with invasive carcinoma, the prognosis will depend upon the invasive component.^[16]^

Many authors recently described the EPC as an encapsulated low-grade invasive carcinoma, and accepted it as a borderline lesion in progression from in situ to invasive carcinoma.^[2,5]^ When an actual invasion is not present, EPC should be evaluated, classified, and managed as an in situ disease; besides, when an invasion is present, the classification and management will be decided according to invasive features.^[17]^

So far, some investigations have reported the metastatic potential of pure EPC and EPC with DCIS/invasion.^[7]^ Furthermore, recommend sentinel lymph node biopsy as an investigative adjunct because it seems to be a practical and prudent way to evaluate the axilla for metastasis.^[18,19]^ The therapeutic management of EPC is controversial and there are no the relevent evidence-based guidelines. In practice, treatment of EPC consists of surgery (wide local excision or mastectomy) ± radiotherapy ± hormonal treatment.^[18]^ Surgical resection is a gold standard for the treatment of papillary lesions, and it is the mainstay of treatment choice during the clinical practice. Breast-conserving surgery and mastectomy (simple, modified radical, or radical) have been utilized in the treatment of EPC. The majority of researches showed that all inclusive cases received any kind of intervention had a good prognosis.^[18,20]^ According to specific selective criteria, radiotherapy, hormonal therapy and chemotherapy may ensue. Adjuvant therapy is a treatment option if DCIS and/or invasion is associated with EPC. Radiotherapy plays an important role in the treatment of young EPC patients. Adjuvant hormonal therapy is also an option for patients who cannot undergo surgery, or patients whose tumors appear repeatedly or age < 50 years.^[6,17]^ Because lacking of the evidence about adjuvant therapy, we based the use of adjuvant therapy on the patient’s opinion after informing them that these treatments are optional.

In a word, the behavior of EPC is very good, as the natures of slow-growth, scarce local relapse, few distant metastases and death owing to breast cancer.^[17]^ Understanding the histological features of this rare tumor and its variant is essential for pathologists, differential diagnosis, tumor staging, adjusting treatment protocol and evaluating the effectiveness. Compared to the previously reported EPC cases of male breast, the lesion of this case was unusually cystic, which making the diagnosis challenging or leading to misdiagnosis as a benign cyst. Therefore, awareness of this unusual feature, repeat biopsy when the pathology result is discordant, and extensive sampling of the lesion are essential for making the correct diagnosis and guiding patient management.

## Acknowledgments

The authors gratefully acknowledge the supports by the Clinical Research Special Funding Fund of Wu Jieping Medical Foundation under Grant number 320.6750.2022-19-80.

## Author contributions

**Methodology:** Zhenya Zhao.

**Resources:** Li Wang.

**Writing – original draft:** Bo wang.

**Writing – review & editing:** Bo wang, Xin Xu.
